# Dendritic Self-assembled Structures from Therapeutic Charged Pentapeptides

**DOI:** 10.1021/acs.langmuir.2c02010

**Published:** 2022-10-13

**Authors:** Karima El Hauadi, Leonor Resina, David Zanuy, Teresa Esteves, Frederico Castelo Ferreira, Maria M. Pérez-Madrigal, Carlos Alemán

**Affiliations:** † Departament d’Enginyeria Química and Barcelona Research Center for Multiscale Science and Engineering, EEBE, Universitat Politècnica de Catalunya, C/ Eduard Maristany 10-14, Barcelona 08019, Spain; ‡ Department of Bioengineering, iBB − Institute for Bioengineering and Biosciences, Instituto Superior Técnico, Universidade de Lisboa, Avenida Rovisco Pais 1, Lisboa 1049-001, Portugal; § Associate Laboratory i4HB—Institute for Health and Bioeconomy at Instituto Superior Técnico, Universidade de Lisboa, Avenida Rovisco Pais 1, Lisboa 1049-001, Portugal; ∥ Institute for Bioengineering of Catalonia (IBEC), The Barcelona Institute of Science and Technology, Baldiri Reixac 10-12, Barcelona 08028, Spain

## Abstract

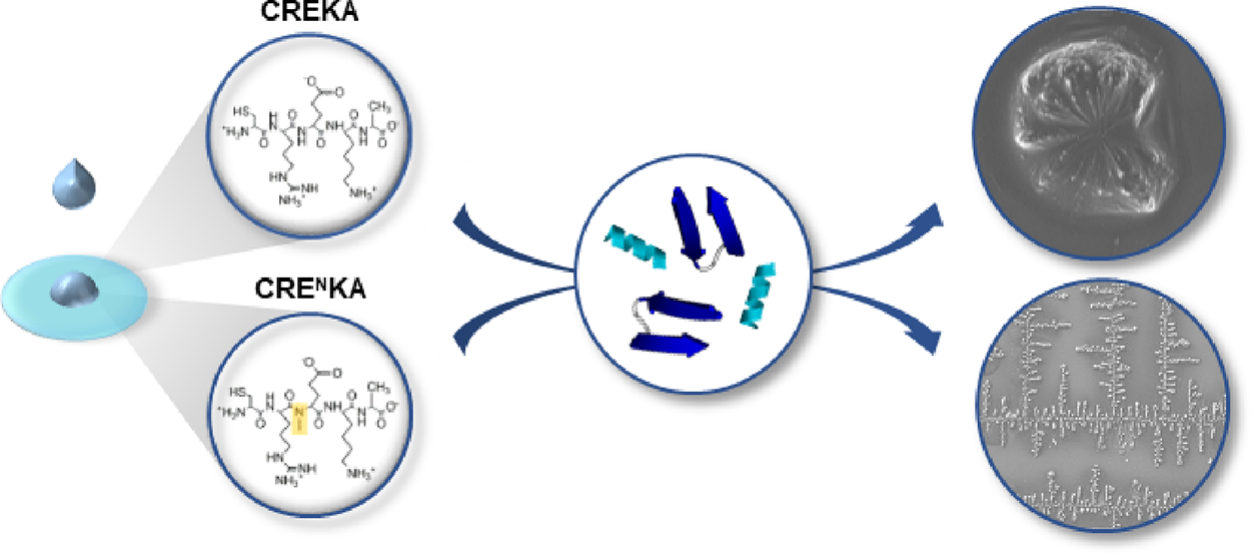

CRE^N^KA [Cys-Arg-(*N*Me)Glu-Lys-Ala, where (*N*Me)Glu refers to *N*-methyl-Glu], an anti-cancer pentapeptide that induces prostate tumor necrosis and significant reduction in tumor growth, was engineered to increase the resistance to endogenous proteases of its parent peptide, CREKA (Cys-Arg-Glu-Lys-Ala). Considering their high tendency to aggregate, the self-assembly of CRE^N^KA and CREKA into well-defined and ordered structures has been examined as a function of peptide concentration and pH. Spectroscopic studies and atomistic molecular dynamics simulations reveal significant differences between the secondary structures of CREKA and CRE^N^KA. Thus, the restrictions imposed by the (*N*Me)Glu residue reduce the conformational variability of CRE^N^KA with respect to CREKA, which significantly affects the formation of well-defined and ordered self-assembly morphologies. Aggregates with poorly defined morphology are obtained from solutions with low and moderate CREKA concentrations at pH 4, whereas well-defined dendritic microstructures with fractal geometry are obtained from CRE^N^KA solutions with similar peptide concentrations at pH 4 and 7. The formation of dendritic structures is proposed to follow a two-step mechanism: (1) pseudo-spherical particles are pre-nucleated through a diffusion-limited aggregation process, pre-defining the dendritic geometry, and (2) such pre-nucleated structures coalesce by incorporating conformationally restrained CRE^N^KA molecules from the solution to their surfaces, forming a continuous dendritic structure. Instead, no regular assembly is obtained from solutions with high peptide concentrations, as their dynamics is dominated by strong repulsive peptide–peptide electrostatic interactions, and from solutions at pH 10, in which the total peptide charge is zero. Overall, results demonstrate that dendritic structures are only obtained when the molecular charge of CRE^N^KA, which is controlled through the pH, favors kinetics over thermodynamics during the self-assembly process.

## Introduction

Cancer is the leading cause of death worldwide, being responsible for nearly 10 million deaths in 2020 (nearly one in six deaths).^1^ Although over the last few years significant progress has been made in cancer treatment, which involves chemotherapy, biological and hormonal therapy, surgery, and/or radiation, the two main problems still persist: the current cancer treatment has a high cost and, what is worse, it still produces adverse side effects. The latter are of particular concern when chemotherapeutic agents are used. For example, doxorubicin, which is a conventional and still widely used chemotherapeutic agent, causes oxidative stress-mediated injury to the brain, kidney, and heart.^2−4^ Furthermore, cancer cells can develop resistance to chemotherapeutic drugs, which results in higher mortality rates.^5,6^

In recent years, therapeutic peptides have become a novel and promising approach for the development of anti-cancer agents with less potential side effects.^7−12^ Anti-cancer peptides (ACPs) exhibit several advantages over chemistry-based chemotherapeutic agents, such as high specificity and low toxicity to normal cells. They also display intrinsic disadvantages, the most remarkable ones being cell membrane impermeability and poor *in vivo* stability.^13^ However, such drawbacks can be partially or, even, totally overcome by designing suitable peptide modifications.

**Scheme 1:**
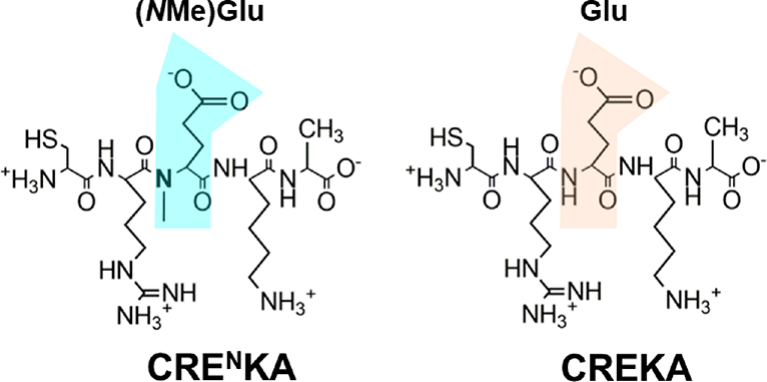
Chemical Structure of CRE^N^KA and CREKA (the (*N*Me)Glu and Glu Residues are Indicated)

Among ACPs, CRE^N^KA [Cys-Arg-(*N*Me)Glu-Lys-Ala, where (*N*Me)Glu refers to *N*-methyl-Glu] was found to be particularly attractive for prostate cancer on account of its small size (*i.e.*, five residues only).^14^ This ACP, which induces prostate tumor necrosis and significant reduction in tumor growth, was inspired by CREKA (Cys-Arg-Glu-Lys-Ala) (Scheme 1), a pentapeptide discovered by the *in vivo* phage display technique^15^ that has been extensively utilized for the image diagnosis of tumors^16−19^ and for inhibition of tumor cell migration and invasion.^14,20^ Furthermore, CREKA and CRE^N^KA have been loaded into intrinsically conducting polymer films and nanoparticles to promote their specificity toward fibrin–fibronectin complexes^21,22^ and to regulate their delivery by electro-stimulation,^23^ respectively. In addition, CREKA has been extensively used for therapeutic applications,^24−28^ including cancer treatment.^24,27,28^ Not only did the substitution of Glu in CREKA by (*N*Me)Glu in CRE^N^KA over-stabilize the peptide bioactive conformation but it also significantly increased its resistance to endogenous proteases.^29^

Recently, we reported that both CRE^N^KA and CREKA tend to rapidly form aggregates, which increased in size, from the nanometric to the submicrometric scale, with peptide concentration,^30^ this behavior being more pronounced for CRE^N^KA than for CREKA. Indeed, the diameter of CRE^N^KA aggregates, as measured by dynamic light scattering, increased from 59 ± 21 to 470 ± 172 nm when the peptide concentration varied from 0.5 to 5 mg/mL, while that of the CREKA ones increased from 255 ± 55 to 589 ± 93 nm. However, many aspects of CREKA and CRE^N^KA aggregates, including their morphologies, remain unstudied.

Peptide aggregates are usually formed by the self-assembly of individual molecules, which under controlled conditions form supramolecular structures through well-defined non-covalent interactions.^31^ For a given peptide, not only does the peptide concentration define the self-assembly process but also the properties of the environment (*i.e.,* polarity and volatility) and, in some cases, the substrate as well.^32^ In order to understand the driving forces that dominate peptide self-assembly and assembly mechanisms, this process has been carefully studied lately for model peptides, including polar,^33−35^ amphiphilic,^36−38^ and highly hydrophobic compounds.^39−41^ Regarding therapeutic peptides, their self-assembly in amorphous or highly ordered aggregates may reduce the physical stability of the peptides in question, leading to not only a loss in activity but also other critical problems, such as toxicity and immunogenicity.^42^ Therefore, within this context, it is worth noting that complete knowledge and understanding of the aggregation tendency of ACPs is of fundamental importance for their clinical usage.

Herein, we aim to investigate, for the first time, the self-assembly of CRE^N^KA ACP and, by extension, of its parent peptide, CREKA. Initially, we provide experimental evidence of the secondary structures preferred by both peptides as a function of peptide concentration and pH, which have been identified in the solution as well as in the aggregate (solid) state using circular dichroism (CD) and FTIR spectroscopy, respectively. Spectroscopic results have been supported by molecular dynamics (MD) computer simulations based on atomistic models. Finally, the experimental conditions that give rise to well-defined self-assembled aggregates have been examined, and the shape of such aggregates has been characterized by SEM. A self-assembly mechanism is proposed to explain the formation of CRE^N^KA dendritic microstructures with fractal geometry.

## Methods

### Materials

CREKA and CRE^N^KA peptides with >98% of HPLC purity were purchased from Biomatik (Toronto, ON). Ultrapure Milli-Q water was used to prepare all the aqueous solutions.

### Sample Preparation

Initial stock solutions of CREKA and CRE^N^KA peptides were prepared at 5 mg/mL concentration using Milli-Q water as the solvent. The peptide concentration was reduced by adding more Milli-Q water to the stock solutions. Solutions at three pH levels (4, 7, and 10) were considered, with acid and basic pH levels being adjusted using concentrated HCl and NaOH solutions, respectively.

### Spectroscopic Studies

CD spectra were recorded between 200 and 250 nm at room temperature using a Chirascan plus qCD equipment, a 10 mm cell path, and 700 μL of aqueous peptide solutions at different concentrations and pH levels. Spectra, which were acquired at a scan speed of 60 nm·min^–1^ with a 1 nm step using a 1 nm bandwidth and a time-per-point of 1 s, were averaged after three accumulations and corrected by subtraction of the background spectrum.

FTIR spectra of solid peptides were recorded on an FTIR Jasco 4100 spectrophotometer equipped with an attenuated total reflection accessory (Top-plate) and a diamond crystal (Specac model MKII Golden Gate Heated Single Reflection Diamond ATR). Samples, which were evaluated using the spectra manager software, were prepared dropping 20 μL of aqueous peptide solution on aluminum foil and left at 4 °C until complete solvent drying. For each sample, 32 scans were recorded between 4000 and 600 cm^–1^ with a resolution of 4 cm^–1^.

### Computer Simulations

All bonding and non-bonding parameters for standard amino acids were obtained from the Amber03 force field.^43^ The parameters of the non-coded (*N*Me)Glu residue had previously been computed and fitted into Amber03.^43^

Two different systems, one for each studied peptide, were built by randomly placing 15 identical molecules in a simulation box with an intermolecular average distance of about 1.8 nm (*i.e.,* molecules were mostly non-interacting among each other, according to the “minimum-bias” approach). Under the simulated conditions (neutral pH), each studied peptide molecule presented a positive net charge, which was neutralized by adding a chloride ion per strand, for a total of 15 anions per studied model. Finally, each simulation box (9.5 × 8.5 × 9.0 nm^3^) was filled with approximately 23,000 TIP3P water molecules,^44^ with overlapping water molecules being removed.

MD series were performed with NAMD 2.10 software package.^45^ The time step was set at 2 fs, and the distances of all bonds involving hydrogen atoms were kept at their equilibrium values with the RATLLE algorithm.^46^ Atom pair distance cut-offs were applied at 1.4 nm to compute all van der Waals interactions. To avoid discontinuities in this energy component, the van der Waals energy term was forced to slowly converge to zero by applying a smoothing factor from 1.0 nm. Electrostatic interactions were extensively computed by means of Ewald summations. The real space term was defined by the van der Waals cut-off (1.4 nm), while the reciprocal space was computed by interpolation of the effective charge into a charge mesh with a grid thickness of 1 point per Å^3^ (particle mesh Ewald).^47^ In all MD simulations, both the temperature and pressure were controlled by the weak coupling method, the Berendsen thermo-barostat,^48^ and a time constant of 1 ps was applied for heat bath coupling and pressure relaxation.

Equilibration was achieved by applying the following steps: (1) the energy of each system was relaxed by 10^4^ steps of energy minimization using the Newton–Raphson method; (2) then, the solvent was equilibrated using a 1 ns-long trajectory with NVT conditions at 500 K while the peptides were kept frozen; (3) the temperature was set at 298 K, and another 1 ns NVT trajectory was run, unfreezing the peptide chains for thermal equilibration; and (4) 1 ns under NPT conditions, the pressure set at 1.034 bar, and keeping the former temperature in order to relax the density of the solution. This later step is the beginning of the production runs of each trajectory series, keeping identical simulation conditions to those of the NPT equilibration cycle.

### Morphological Studies

Twenty microliters of aliquots of the peptide solutions at different concentrations and pH values was placed on glass coverslips and kept inside a cold chamber (4 °C) until dryness (∼15 days). Scanning electron microscopy (SEM) studies were performed in a Focussed Ion Beam Zeiss Neon 40 scanning electron microscope operating at 5 kV and equipped with an EDX spectroscopy system. Samples were mounted on a double-sided adhesive carbon disk and sputter-coated with a thin layer of carbon to prevent sample charging problems.

## Results and Discussion

### Secondary Structure in the Solution and Solid State

First, the conformation of CREKA and CRE^N^KA was examined in solution by CD considering not only different peptide concentrations (from 0.01 to 1 mg/mL) but also diverse pH values (4, 7, and 10). Indeed, their secondary structure, as well as the tendency to self-assemble into ordered structures, was expected to be drastically affected by the ionization state of the charged residues: Arg, Glu/(*N*Me)Glu, and Lys residues (Scheme 1). The p*K*_a_ values of Glu, Arg, and Lys side groups are 4.2, 12.5, and 10.5, respectively; while the p*K*_a_ values of the ionizable amino and carboxytale groups of the N- and C-terminus are ∼8 and ∼3, respectively.^49^ Accordingly, the Glu/(*N*Me)Glu side group will be predominantly neutral at pH 4, while the Arg and Lys side groups and the two backbone terminal groups will remain ionized (total molecular charge: +2). Instead, all such residues and backbone terminal groups will be predominantly ionized at pH 7 (total molecular charge: +1), while at pH 10, the amino terminal group will be deionized (total charge: +0). Hence, the pH will govern ionization and, thus, the intramolecular and intermolecular electrostatic interactions that control the secondary structure of CREKA and CRE^N^KA.

**Figure 1:**
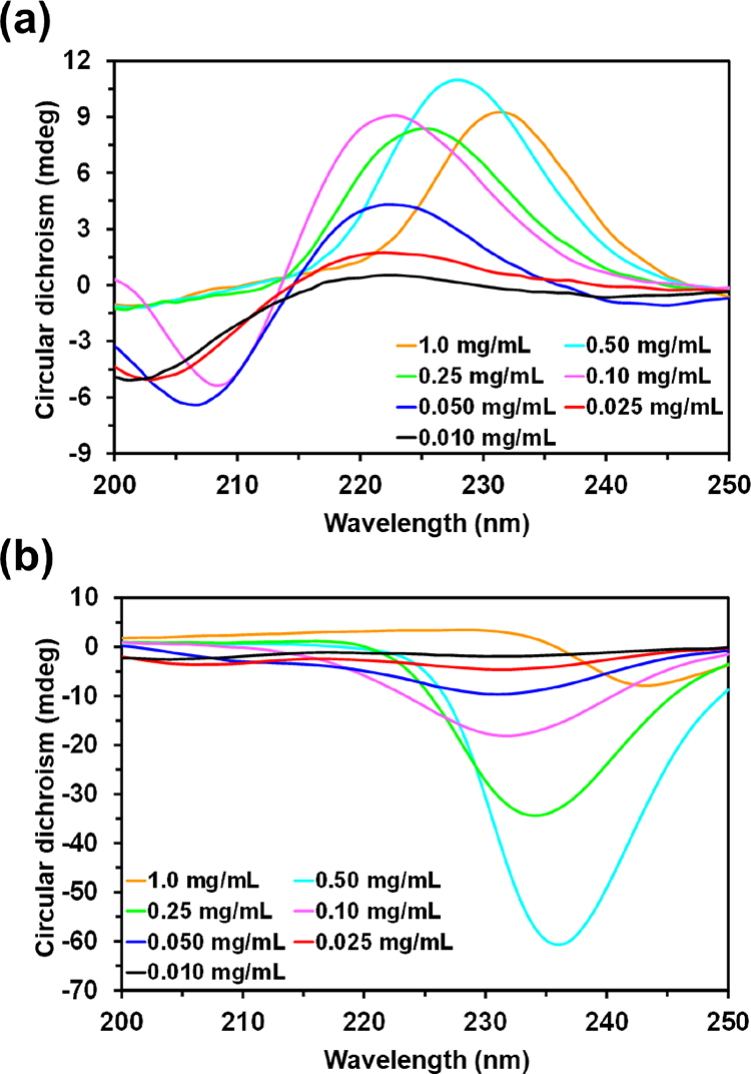
CD spectra for (a) CREKA and (b) CRE^N^KA in aqueous solution and pH 7 at different peptide concentrations.

The CD spectra recorded for different peptide solutions at neutral pH are displayed in Figure 1. For concentrations ≤0.1 mg/mL, CREKA exhibits a negative band at around 200 nm, which shifts to a higher wavelength with an increasing peptide concentration (208 nm at 0.1 mg/mL), and a positive band at 222 nm (Figure 1a). This profile, which is maintained at pH 4 and 10 (Figure S1a), is fully consistent with a random structure. The shape of the spectrum changes when the CREKA concentration is ≥0.25 mg/mL. In such cases, a single positive band (*i.e.,* the negative band disappears) is detected, the position of the maximum increasing with the peptide concentration (from 225 nm for 0.25 mg/mL to 232 nm for 1.0 mg/mL). These spectra, which are typically associated with a β-turn,^50−52^ are in good agreement with the bioactive conformation proposed for CREKA, which consisted of a turn conformation with the charged side chains pointing outward to facilitate the formation of intermolecular interactions.^53^

The spectra obtained for CRE^N^KA were completely different from those recorded for CREKA. CRE^N^KA exhibits a single negative band that shifts from 231 nm at low concentrations to 243 nm at 1.0 mg/mL (Figure 1b). Those spectra have been related with β-sheets, and the shift observed at increasing peptide concentration, which is practically independent of the pH (Figure S1b), suggests the enhancement of the β-sheet structure.^54,55^ Accordingly, it is hypothesized that intermolecular CRE^N^KA···CRE^N^KA interactions favor a more extended structure, which should promote self-assembly aggregation processes. Our hypothesis is supported by the conformational restrictions imposed by the (*N*Me)Glu residue, which stabilize elongated conformations.^29^

**Figure 2:**
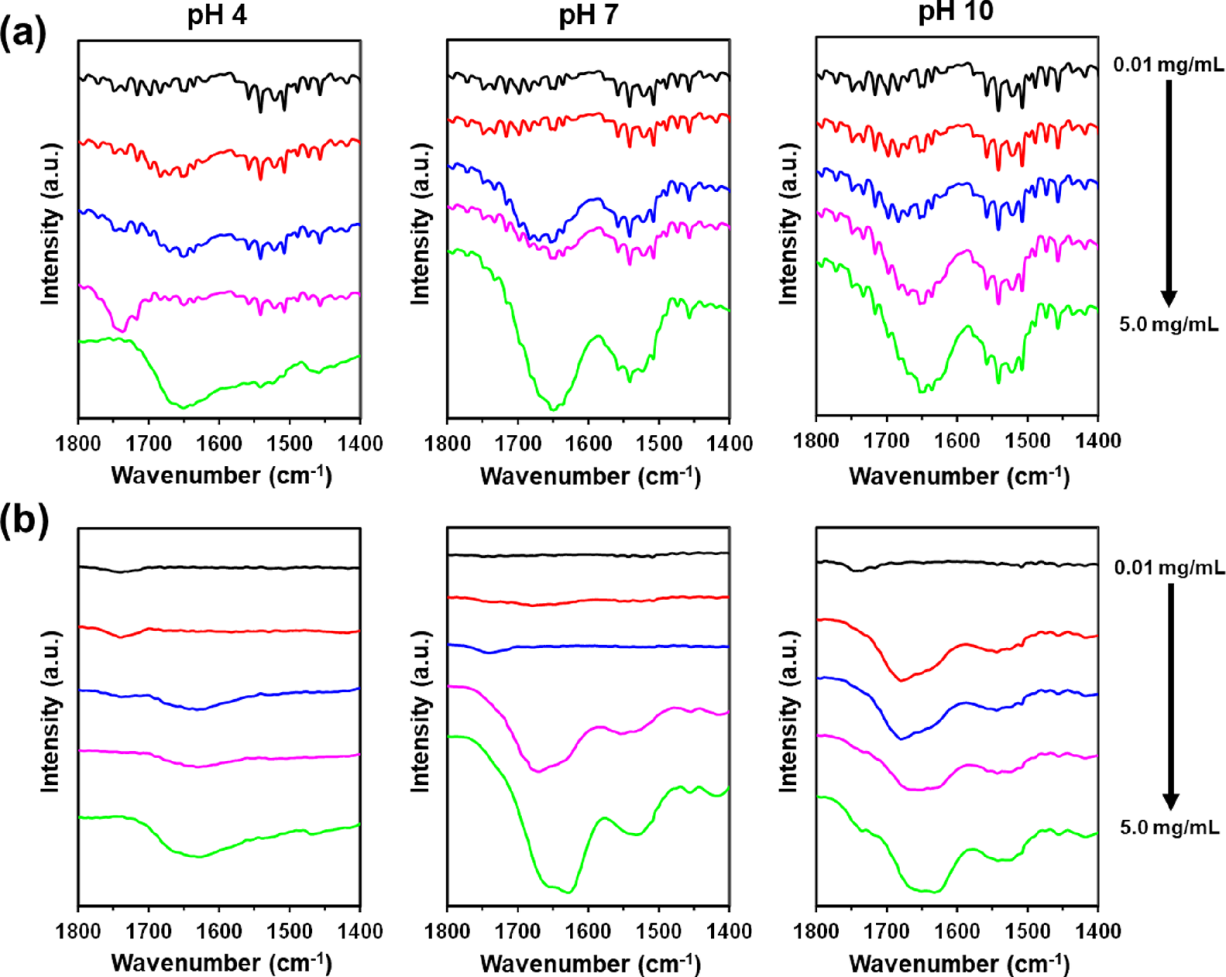
FTIR spectra in the amide I and II regions for (a) CREKA and (b) CRE^N^KA peptides. Spectra were recorded after evaporation of the solvent at 4 °C. For this purpose, peptide aqueous solutions at 0.01, 0.1, 1.0, 2.0, and 5.0 mg/mL concentrations and at pH 4, 7, and 10 were dropped on an aluminum substrate.

The secondary structure of CREKA and CRE^N^KA in the aggregate state was studied as a function of the ionization state and peptide concentration of the feeding solution using FTIR spectroscopy. For this purpose, 20 μL of peptide aqueous solutions at 0.01, 0.1, 1.0, 2.0, and 5.0 mg/mL concentrations and at pH 4, 7, and 10 were dropped on an aluminum substrate and left at 4 °C until complete solvent evaporation. Figure 2 displays the recorded spectra in the region of the amide I (1600–1800 cm^–1^) and amide II (1470–1570 cm^–1^), which are the most prominent and sensitive bands of the peptide backbone and are related to peptide secondary structural components.

For both peptides, the spectra recorded at neutral and basic pH are better defined than those at acid pH, which suggests that electrostatic interactions play a crucial role in the aggregation process. CREKA shows a predominant broad adsorption band centered at 1650 cm^–1^, which increases with peptide concentration (Figure 2a). Although this has been assigned to a random coil conformation, the small peaks in the range of 1600–1700 cm^–1^ suggest that other secondary structural motifs have a minor contribution (*e.g.,* β-sheet, 3_10_-helix, and β-turn at 1620, 1669, and 1683 cm^–1^, respectively). This feature is fully consistent with the fact that small peptides present more than one conformation since subtle conformational rearrangements allow the interconversion between different secondary structures. Conversely, small peaks are not detected in the CRE^N^KA spectra, which only show a pronounced broad band centered at the 1620–1660 cm^–1^ interval (Figure 2b). At the higher concentrations and pH 7 and 10, this band splits into two peaks centered at 1628 and 1658 cm^–1^, which are consistent with pseudo-extended and turn or random coil structures, respectively. In CRE^N^KA, the conformational variability of CREKA is expected to be restricted by the constrictions imposed by the substitution of Glu by (*N*Me)Glu. In addition, the amide II peak, which corresponds to the N–H bending vibration and the C–N stretching vibration (amide II), experiences a redshift from 1550 to 1534 cm^–1^ with increasing peptide concentration, which is consistent with the formation of intermolecular hydrogen bonds in the aggregates.

**Figure 3:**
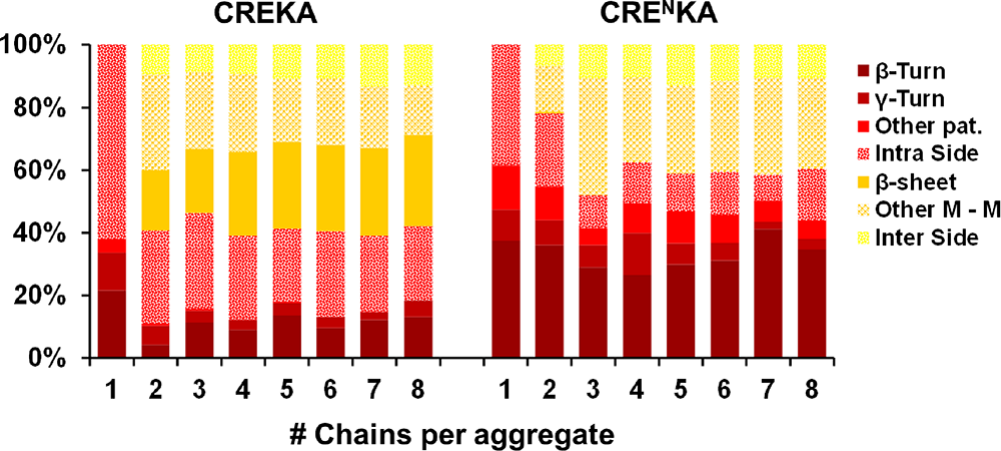
Percentage of types of hydrogen bond patterns as a function of the aggregate size after 70 ns of simulation. Aggregates of one chain refer to chains that are not part of any assembly. In the legend: β*-*Turn and γ*-*Turn refer to the hydrogen bond pattern proper to those conformations, Other pat*.* refers to other intramolecular hydrogen bonds pattern between amide groups, Intra Side to intramolecular hydrogen bonds in which side chain groups are involved, *β-*sheet to intermolecular hydrogen bonds between main chain amide groups in chains adopting β-strand conformations*,* M-M to intermolecular hydrogen bonds between main chain amide groups in strands with not defined conformation, and Inter Side to intermolecular hydrogen bonds in which side chain groups are involved.

Computer MD simulations on CREKA and CRE^N^KA atomistic models were performed considering 15 independent peptides molecules, which were initially non-interacting, in a simulation box filled with water molecules (see the Methods section). After 70 ns of production trajectories, the preferred hydrogen bond patterns were analyzed as a function of how the peptide chains are organized (*i.e.,* isolated or part of an aggregate). The geometric criteria used to account hydrogen bonds were (1) distances H···O shorter than 0.3 nm and (2) angles ∠N–H···O higher than 120.0°. Figure 3 shows the distribution of the preferred hydrogen bonding patterns as a function of the number of chains present in an aggregate for the production trajectory (being number of chains 1 when a peptide is not part of an aggregate). As it can be seen, results confirmed the previously presented observations.

When CREKA chains are not part of an aggregate, they majorly adopt undefined conformations, with predominance of structures that are stabilized by hydrogen bonds involving polar and charged groups from the side chains, whereas CRE^N^KA peptides predominantly present hydrogen bond patterns compatible with defined turn conformations. The behavior of both peptides when aggregating is also differential. Hydrogen bonds in CREKA aggregates are predominantly between main chain amide groups with a clear tendency to form β-sheet patterns, especially when the size of the aggregate (in number of interacting chains) increases. On the other hand, CRE^N^KA aggregates present a strong tendency to not interact via main chain amide groups and, thus, strongly retain the intramolecular hydrogen patterns that were predominant when not being part of an aggregate. While CREKA clusters of chain tend to form hydrogen bond organizations compatible with β-sheet structures, CRE^N^KA clusters tend to organize via other interactions (generally by salt bridges) while preserving the conformational features that they presented before aggregating. Overall, results derived from atomistic modeling using MD simulations are in good agreement with CD and FTIR observations in terms of interactions.

**Figure 4:**
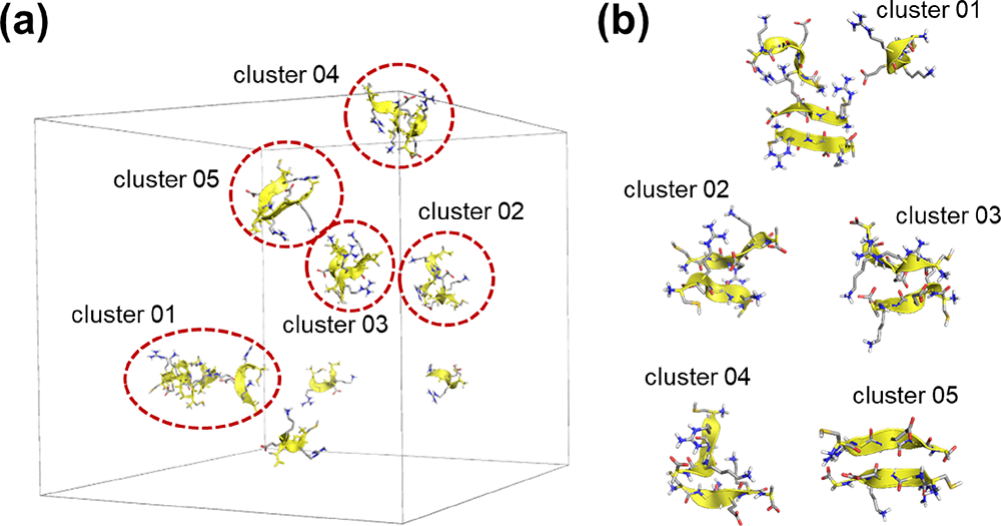
Molecular representation of the last snapshot of the simulated CREKA system: (a) complete simulation box, including non-associated chains, and (b) enlarged detail images of each detected molecular assemblies. Atom colors follow the CPK convention. Hydrophobic hydrogen atoms have been removed for clarity. Main-chain α-carbons and the backbone have been remarked in yellow color.

On the other hand, several interesting structural differences can be observed between both studied peptides. CREKA chains, as statistical analysis had already shown, have an acute tendency to laterally assemble via their amide groups, forming chain pairs. This pattern, which is present in all detected aggregates, corresponds to arrangements compatible with the β-sheet motif, in several degrees of formation. Figure 4 depicts the final snapshot of the simulation, demonstrating that there are 10 chains out 15 participating in aggregated structures. Eight of such chains form either full fletched sheets or structures reminiscent of such organization. Among the five detected clusters, two of them are almost canonic β-sheets (cluster 01 and cluster 05 in Figure 4b), whereas the remaining present assemblies that can be understood as distorted sheets or not fully formed structures.

Two other remarkable features can be observed. First, only one aggregate (cluster 01, Figure 4b) presents one chain whose assembly is not directly driven by the interaction between main chain amide groups. The four chain assemblies show a two-stranded sheet interacting with two extra chains via both salt bridges and dipole-charge interactions. This feature becomes relevant when compared with the aggregation features of the CRE^N^KA system (see below). The second noteworthy aspect is the strand orientation within sheets. Of five two-stranded assemblies, three of them are antiparallel and two are parallel. This structural diversity has been observed in many amyloid-like structure fibers derived from small peptides^56^ when the final outcome of the fully formed fiber depends not only on the inner stability of the sheets themselves but also on the ability to favor both intrasheet long-distance interactions and the possibility of enhancing intersheet lateral interactions.

**Figure 5:**
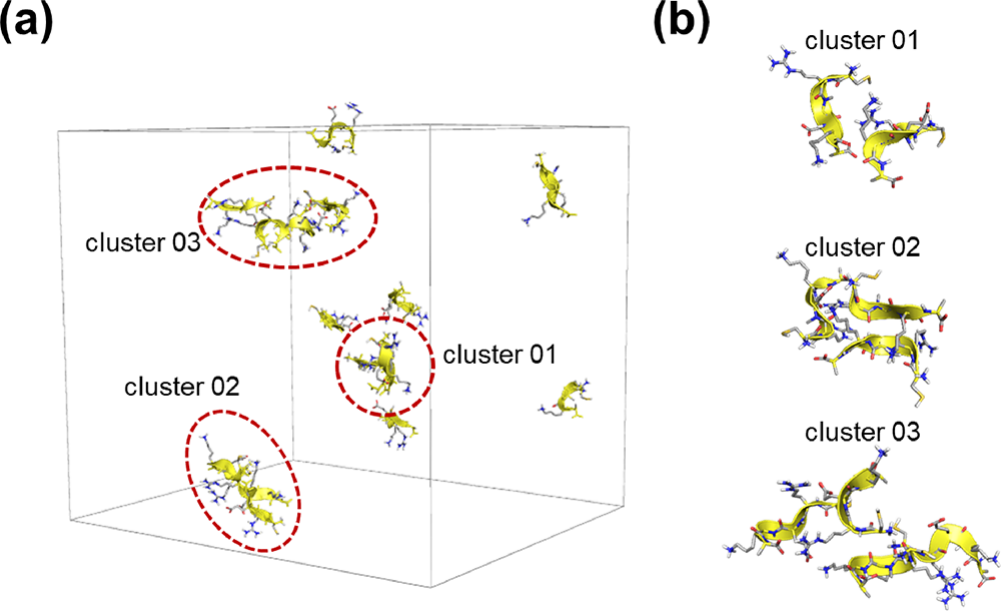
Molecular representation of the last snapshot of the simulated CRE^N^KA system: (a) complete simulation box, including non-associated chains, and (b) enlarged detail images of each detected molecular assemblies. Atom colors follow the CPK convention. Hydrophobic hydrogen atoms have been removed for clarity. Main-chain α-carbons and the backbone have been remarked in yellow color.

In contrast, CRE^N^KA shows significant differences in the assembly outcome compared to the unmodified peptide (Figure 5). Despite presenting a similar ratio of assembled chains versus free strands, the aggregation pattern is quite dissimilar. In CRE^N^KA, the presence of sheet-like structures is reduced to a single cluster (cluster 02 in Figure 5b) even though this structure is not an ideal β-sheet because the presence of a methylated amide group per strands hampers the formation of more than a single interchain hydrogen bond. Moreover, even in this case, a recurrent new structural pattern can be observed, which will be repeated in the other observed assemblies. The most favored interaction pattern consists of polar- and charged-driven interaction between the peptides side chains, which favors the formation of 2D assemblies as a core organization rather than a single rotation axis observed in fibers based on stacking of β-strands. Within this context, cluster 03 becomes exemplary, in which four strands mainly associate via side chain-driven interactions and the core of the aggregate is made up of two strands preserving a turn conformation, which is the predominant arrangement when chains are not part of an aggregate. This crossed-like organization hints a possible growth path compatible with the formation of the fractal flat spikes observed by electronic microscopy. When this new structural trait is combined with previous analysis, in which most CRE^N^KA free chains in the simulation consistently adopted turn conformations, it is possible to infer a potential mechanism of assembly based on lateral association of pre-conformed chains via polar/charge interactions of their respective side chains.

### Morphology of Self-assemblies

The morphology of the self-assemblies formed by CREKA and CRE^N^KA was investigated using SEM and considering different peptides solutions, which were prepared varying both the concentration (from 0.01 to 5.0 mg/mL) and the pH (4, 7, and 10). For this purpose, a drop of 20 μL of peptide solution was placed on a clean glass cover slip and dried at 4 °C until complete desiccation. SEM micrographs displayed in this work correspond to reproducible and abundant morphologies.

The tendency of CREKA to form self-assemblies with well-defined and reproducible morphologies was extremely poor. In fact, pseudo-regular aggregates were only systematically formed using low and moderate peptide concentrations at acidic pH or using low peptide concentrations at neutral pH. In addition, as is illustrated by representative SEM micrographs (Figure S2), the morphologies obtained under such conditions, which predominantly consist of fibers of micrometric thickness, were poorly defined. The absence of reproducible self-assembled nano- and microstructures with well-defined morphology is fully consistent with the large conformational variability observed for CREKA by FTIR spectroscopy (Figure 1), CD (Figure 2), and MD simulations (Figure 3). This conformational variability, together with the fact that intermolecular interactions among CREKA molecules are dominated by strong repulsive and attractive electrostatic interactions, results in a self-assembly process controlled by kinetics instead of thermodynamics: the rapid formation of aggregates stabilized by unspecific interactions prevent the formation of well-defined morphologies. Such behavior opposes that observed for highly aromatic small peptides, which tend to form micro- and nanostructures with ultra-well-defined morphologies stabilized by specific intermolecular interactions (*e.g.,* π–π stacking).^32,39,40^ Hence, those interactions, which are much weaker than electrostatic ones, favor the thermodynamics control over the kinetics control in the self-assembly process.

**Figure 6:**
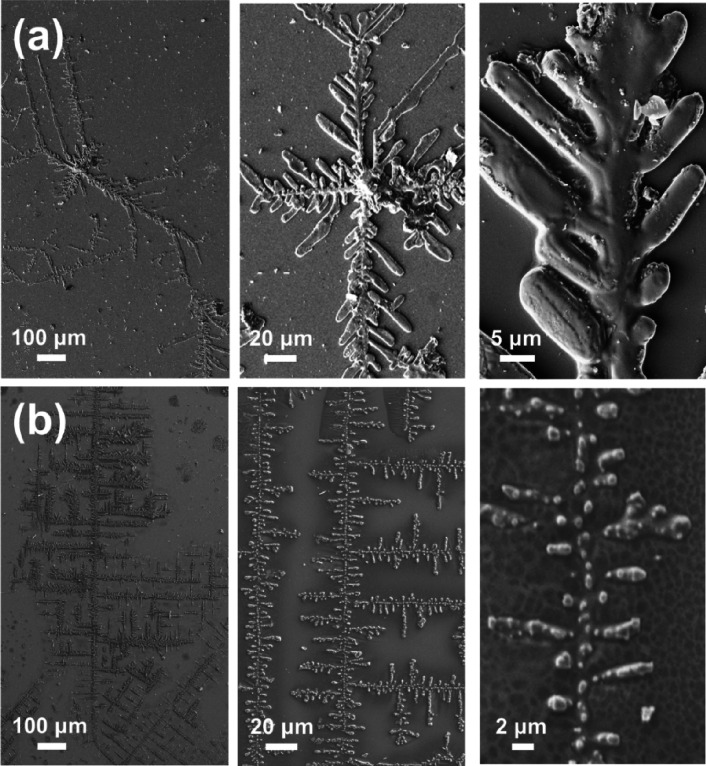
SEM micrographs of dendritic microstructures formed from CRE^N^KA solutions at 4 °C using the following conditions: (a) 0.01 mg/mL at pH 4 and (b) 0.1 mg/mL at pH 7.

In contrast, CRE^N^KA showed a significant tendency to form ordered microstructures when the peptide concentration was low or, even, moderate (≤2 mg/mL). At low peptide concentrations, CRE^N^KA spontaneously formed stable branched dendritic structures with micrometric branches growing from elongated primary frameworks of millimeter length (Figure 6). Such kinds of structures, which exhibit fractal characteristics, were found to be very abundant and repetitive at 0.01 mg/mL at pH 4 (Figure 6a) and 0.1 mg/mL at pH 7 (Figure 6b). Similar self-assemblies were also reported for human amylin,^57^ a small (37 residues) and intrinsically disordered protein, short amphiphilic peptides (*e.g.,* Fmoc-phenylalanine-tyrosine-phosphate),^58^ and highly aromatic peptides (*e.g.,* phenylalanine-oligomers capped with fluorenylmethoxycarbonyl and fluorenylmethyl esters at the N-terminus and C-terminus, respectively),^40^ among others.

In the case of CRE^N^KA, it should be emphasized that the formation of fractal-like structures is pH- and concentration-dependent and only occurred when the net charge of the peptide molecule is the highest or very high and, simultaneously, the peptide concentration is low*.* As discussed above, the neutral state of the side carboxylate group of the (NMe)Glu at pH 4 results in a net peptide charge of +2, while the net charge decreases to +1 at pH 7. Under such conditions, repulsive intermolecular interactions result in a fractal self-assembly through a diffusion-limited aggregation process when the peptide concentration is low enough. The diffusion-limited kinetics is supported by the conformational restrictions induced by (NMe)Glu residues, which drastically reduce the degree of freedom of CRE^N^KA in comparison to CREKA.^29^ Instead, no dendritic-like or any other ordered assembly was detected when the peptide charge is null at pH 10. In addition, low peptide concentrations allow attractive peptide···peptide interactions to dominate the aggregation process, facilitating the orderly self-assembly of molecules. On the contrary, when the peptide concentration is excessively high, the intermolecular separation between functional groups with charges of the same sign is too short and the influence of repulsive peptide···peptide interactions predominates, governing the self-assembly process and leading the molecules to aggregate disorderly.

**Figure 7:**
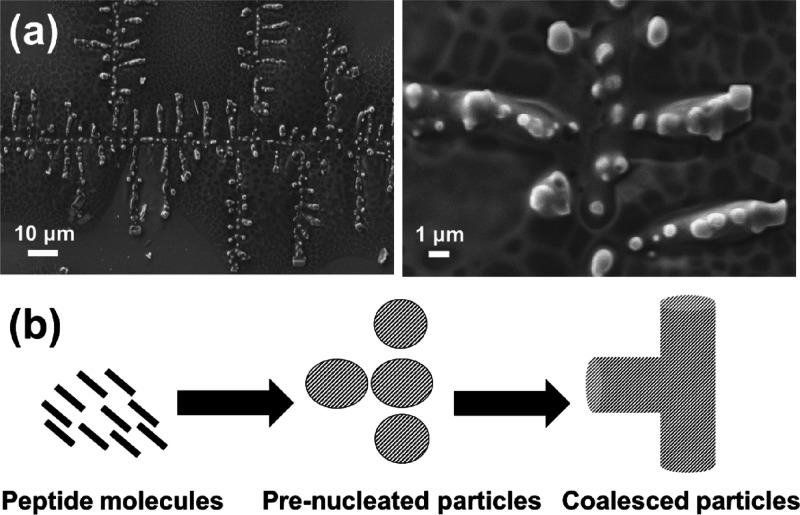
(a) SEM micrographs of dendritic microstructures pre-formed from 0.1 mg/mL CRE^N^KA solutions at pH 7 and 4 °C. Micrographs were recorded when only half of the solvent had slowly evaporated in the cold room. (b) Sketch of the mechanism proposed for the formation of the dendritic structures.

Additional experiments were performed by interrupting the growth of the branched dendritic structures and observing their morphology before the slow evaporation of the solvent ended. Representative SEM micrographs are reported in Figure 7a. As it can be seen, the structures displayed in Figures 6 and 7a are consistent with a two-step self-assembly mechanism, which is sketched in Figure 7b. First, pseudo-spherical particles are pre-nucleated through a diffusion-limited self-assembly process. This step gives place to an interrupted structure with dendritic geometry. After that, the interactions between the solvent-accessible surface of such pre-nucleated structures and the charged/polar groups moieties of peptide molecules in the solution cause more aggregation and the coalescence of pre-nucleated particles (Figure 7c). Therefore, the interrupted dendritic structure transforms into a continuous structure. Details of how the coalescence of neighboring particles occurs are shown in Figure S3, which displays SEM micrographs of structures formed before the solvent was completely evaporated. The space between the particles was filled through the self-assembly of more peptide molecules, which caused the pre-formed particles to grow until they came into contact and merged. The directional growing of the coalescent interparticle assemblies and, consequently, the formation of a continuous fractal geometry have been attributed to the loss of conformational freedom induced by the (*N*Me)Glu residue.

Unfortunately, no additional morphological information could be obtained from the MD simulations. This is due to the limitations of the MD simulations, which, on the one hand, only contain 15 peptide molecules (hundreds, if not thousands, would be needed to establish a correlation with the experimentally observed morphology), and on the other hand, the time scale of the simulations was too short. Although nowadays it is possible to carry out simulations of a few microseconds, the observed self-assembly process is not controlled kinetically but thermodynamically and therefore occurs at much higher time scales. Finally, it should be mentioned that, as the self-assembly occurs at the same time as the evaporation of the solvent, the incorporation of this last process to the simulation would greatly complicate it.

Because of their unusualness, no practical application has been developed yet for peptide-based self-assembled dendritic microstructures with fractal patterns. However, the exploitation of such hierarchical architectures in applications requiring multiple-length scale is expected to be valuable in the near future. The unique properties of fractal dendritic structures, as for example the large surface area and the self-similarity, combined with the advantages of peptides as biomaterials are beneficial for potential applications in advanced biosensors, microprinting, biocatalysis, and, in general, in the biomedical field. In the case of the studied ACP, CRE^N^KA, it is not yet known whether the dendritic-assembled structures that we have observed *in vitro* are stable *in vivo*, in which the surrounding conditions are much less controlled. However, the exploitation of the fractal geometry to increase the efficacy of the ACP by adjusting a sustained dosage deserves consideration as it would reduce the adverse side effects. Therefore, future studies will focus on examining the stability of CRE^N^KA assemblies under physiological conditions and in controlling their disassembly process.

**Figure 8:**
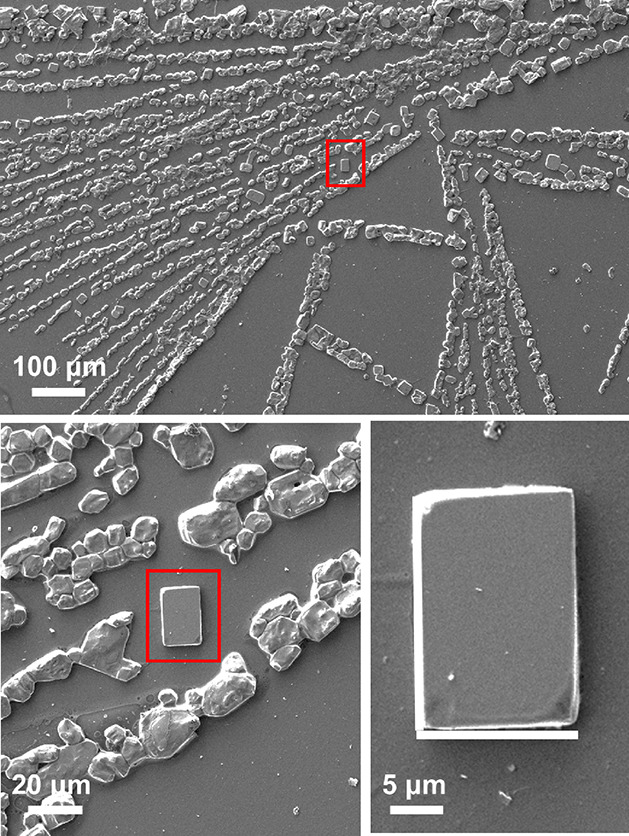
SEM micrographs of rhombohedrum crystals pre-formed from 0.1 mg/mL CRE^N^KA solutions at pH 7 and 4 °C. Micrographs were recorded when only half of the solvent had evaporated.

Another interesting feature consisted in the formation of micrometric (∼15 × 20 μm^2^) rhombohedrum crystals (six faces) that were sporadically detected in the dendritic microstructures pre-formed from 0.1 mg/mL CRE^N^KA solutions at neutral pH (Figure 8). However, although these structures were clearly identified when the self-assembly process was interrupted, they were never detected when the interparticle space was filled by the peptide at the end of the self-assembly process. These results evidence that the conformational restrictions imposed in CRE^N^KA contribute to a crystallization process, even though crystals were not abundant enough and were too small for crystal structure determination using X-ray diffraction.

**Figure 9:**
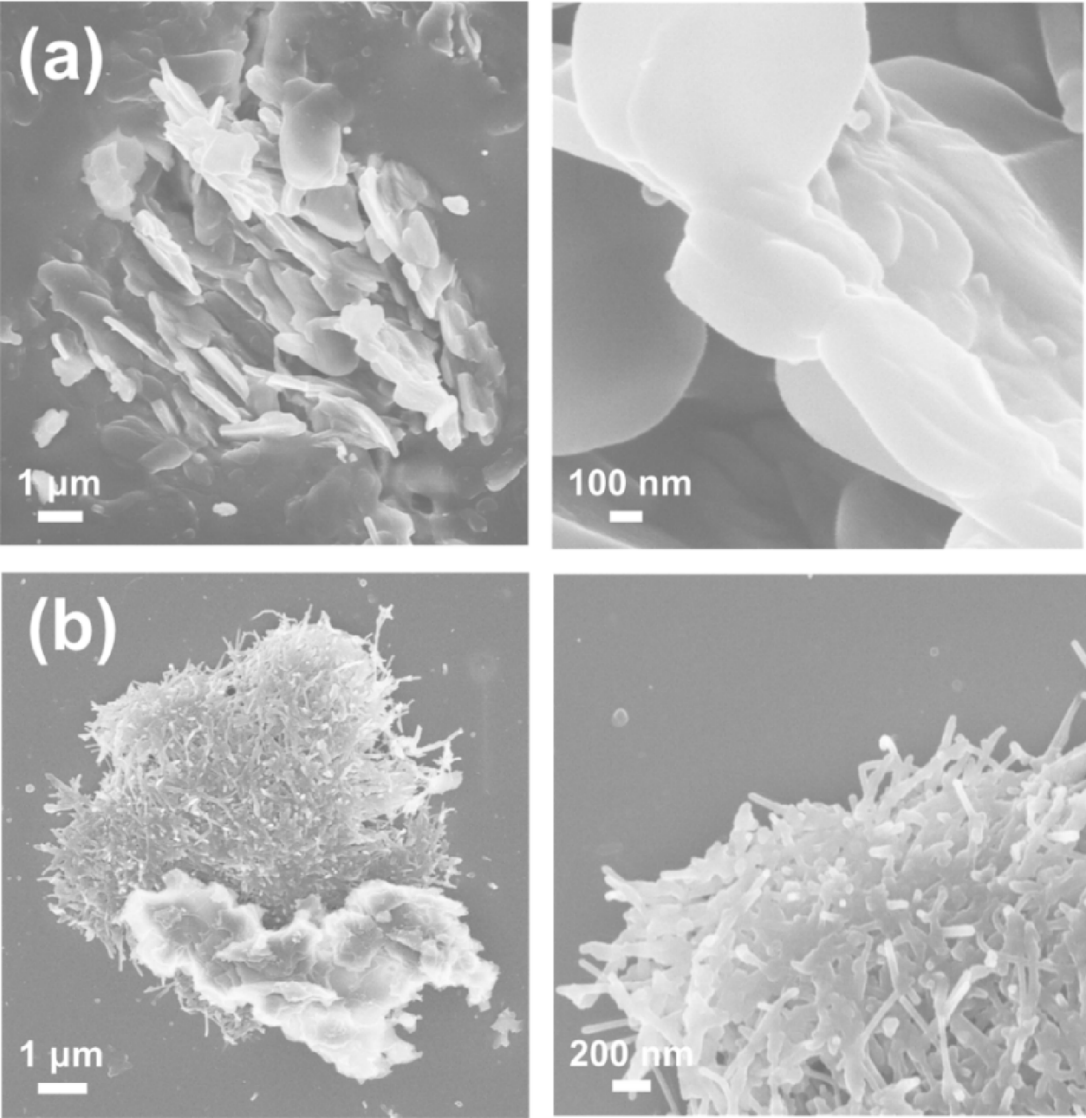
SEM micrographs of irregular particles made of (a) nanoplates and (b) nanofibers obtained for 1.0 mg/mL CRE^N^KA solutions at pH 7.

Finally, at higher peptide concentrations, CRE^N^KA self-assembled into irregular microparticles that consisted of densely packed nanoplates or nanofibers (*i.e.,* plates of micrometric length and nanometric thickness). These structures, which are illustrated in Figure 9 for the 1.0 mg/mL peptide solution at pH 7, suggest a change in the self-assembly mechanism, which is hypothesized as the growth of abundant nanostructures around a nucleation site. However, the thickness of such nanostructure is apparently limited by peptide–peptide electrostatic interactions, which are expected to have a greater role than in solutions with lower peptide concentrations. Similar assemblies, especially those of clustered nanofibers, were also observed for 2.0 mg/mL peptide solutions (Figure S4). However, although the size and density of nanofibers were similar to those observed for 1.0 mg/mL peptide solutions, the global size of the irregular particles was slightly higher. Instead, no regular self-assembly was detected for 5 mg/mL CRE^N^KA solutions, regardless of pH value. At such a high peptide concentration, repulsive interactions clearly govern the dynamics of the CRE^N^KA molecules in solution and therefore, the evaporation of the solvent resulted in the deposition of irregular peptide layers on the glass substrate.

In summary, results suggest that the aggregation of the two studied peptides is due to intermolecular electrostatic interactions among their ionized groups. Such aggregation phenomena are observed for both CREKA and CRE^N^KA, which is consistent with the fact that both bear the same ionizable side chains. On the other hand, the conformational restriction introduced in CRE^N^KA seems to play a major role in the organization of the peptide molecules during the aggregation process. While CREKA have a poor tendency to form aggregates with well-defined morphologies, the conformational restriction imposed in CRE^N^KA is consistent with the formation of microstructures with well-defined shapes. In particular, the formation of dendritic microstructures with fractal geometry, which are observed for diluted CRE^N^KA solutions at acid and neutral pH, is consistent with the regular nucleation process (*i.e.*, the lateral association of pre-conformed chains) identified by MD simulations.

## Conclusions

Harnessing the self-assembly of ACPs for a more efficient release is a significant challenge to improve the efficacy of cancer treatments and eliminate toxicity in healthy tissues. In this work, we have reported the self-assembly tendencies of CRE^N^KA, an ACP with proved efficacy, and its parent compound, CREKA. Conformational studies in solution and in the aggregated states have been conducted using CD, FTIR spectroscopy, and MD simulations, which reveal that the restrictions imposed by the (*N*Me)Glu residue drastically reduce the flexibility of CRE^N^KA in comparison to CREKA. Apparently, this feature is crucial to explain the significant differences found between the self-assembly behavior of the two peptides. Also, the net molecular charge, which is controlled through the pH, is the key for the formation of aggregates with well-defined, regular, and reproducible morphologies.

CREKA, which rarely self-assembles into aggregates with well-defined morphologies, tends to form non-shaped structures with no regular organization. Instead, CRE^N^KA forms abundant and reproducible dendritic microstructures with fractal geometry when the following conditions are fulfilled: (1) the net charge of the peptide is +2 or +1 (acid and neutral pH), which, in conjunction with the conformational restrictions, favors ordered self-assembly, and (2) the peptide concentration in the solution is low enough to avoid that peptide–peptide repulsive interactions that dominate the dynamics of the solution. Furthermore, we have observed that dendritic microstructures grow in a two-step process: (1) formation of pre-nucleated pseudo-spherical particles and, even, rhombohedrum crystals and (2) filling of the interparticle space following a directional self-assembly process. We hope that this ground work facilitates further research regarding the therapeutic utilization of ACPs, as for example the encapsulation of CRE^N^KA in micro- and nanocarriers for controlled targeted delivery in which we are currently working on.

## Supporting Information Available

CD spectra and SEM micrographs of microstructures and irregular particles that formed on CREKA and CRE^N^KA solutions (PDF)

## Notes

In Memoriam of Eric A. Perpète, a colleague, collaborator, and friend who passed away recently.
