# Supplementary material for: Dendritic Self-assembled Structures from Therapeutic Charged Pentapeptides
Source: Langmuir. 2022 Oct 13;38(42):12905–14. doi: 10.1021/acs.langmuir.2c02010 (PMC9988208; doi:10.1021/acs.langmuir.2c02010)
Supplement: Supplementary file 1 — la2c02010_si_001.pdf [file la2c02010_si_001.pdf]

SUPPORTING INFORMATION

Dendritic Self-Assembled Structures from Therapeutic Charged Pentapeptides

Karima El Hauadi,[a,♦] Leonor Resina,[a,b,c,♦] David Zanuy,[a] Teresa Esteves,[b] Frederico Castelo Ferreira,[b,c] Maria M. Pérez-Madrigal,[a,*] and Carlos Alemán[a,d,*]

[a] Departament d'Enginyeria Química and Barcelona Research Center for Multiscale Science and Engineering, EEBE, Universitat Politècnica de Catalunya, C/ Eduard Maristany 10-14, 08019, Barcelona, Spain

[b] iBB – Institute for Bioengineering and Biosciences, Department of Bioengineering, Instituto Superior Técnico - Universidade de Lisboa, Avenida Rovisco Pais 1, 1049-001 Lisboa, Portugal

[c] Associate Laboratory i4HB—Institute for Health and Bioeconomy at Instituto Superior Técnico, Universidade de Lisboa, Avenida Rovisco Pais 1, 1049-001 Lisboa, Portugal

[d] Institute for Bioengineering of Catalonia (IBEC), The Barcelona Institute of Science and Technology, Baldiri Reixac 10-12, 08028 Barcelona Spain

♦ These authors contributed equally to this work

* Correspondence to m.mar.perez@upc.edu and carlos.aleman@upc.edu

Page S2	Figure S1. CD spectra for peptides in aqueous solution and pH 4 and 10.
Page S3	Figure S2. SEM micrographs of microstructures formed from CREKA solutions.
Page S4	Figure S3. SEM micrographs of microstructures formed from CRE^{N}KA solutions.
Page S5	Figure S4. SEM micrographs of irregular particles formed from CRE^{N}KA solutions.

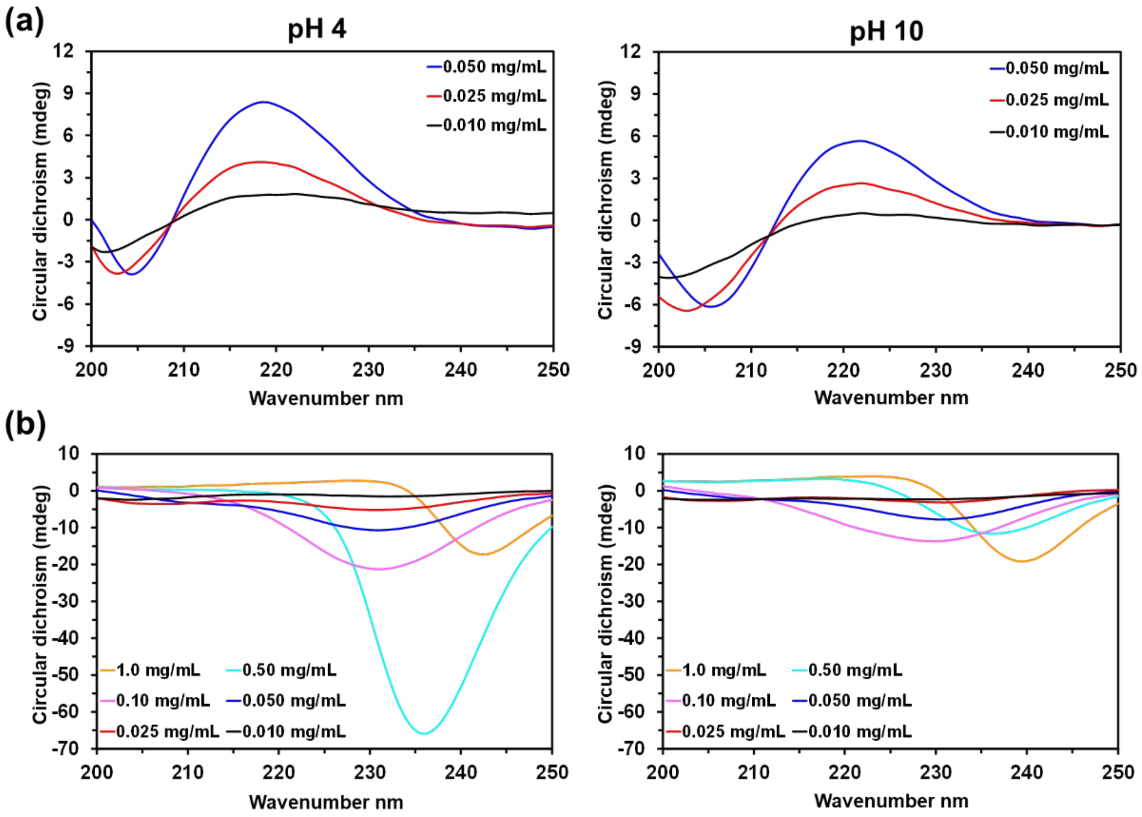

Figure S1. CD spectra for (a) CREKA and (b) CRE^{N}KA in aqueous solution and pH 4 (left) and pH 10 (right) at different peptide concentrations.

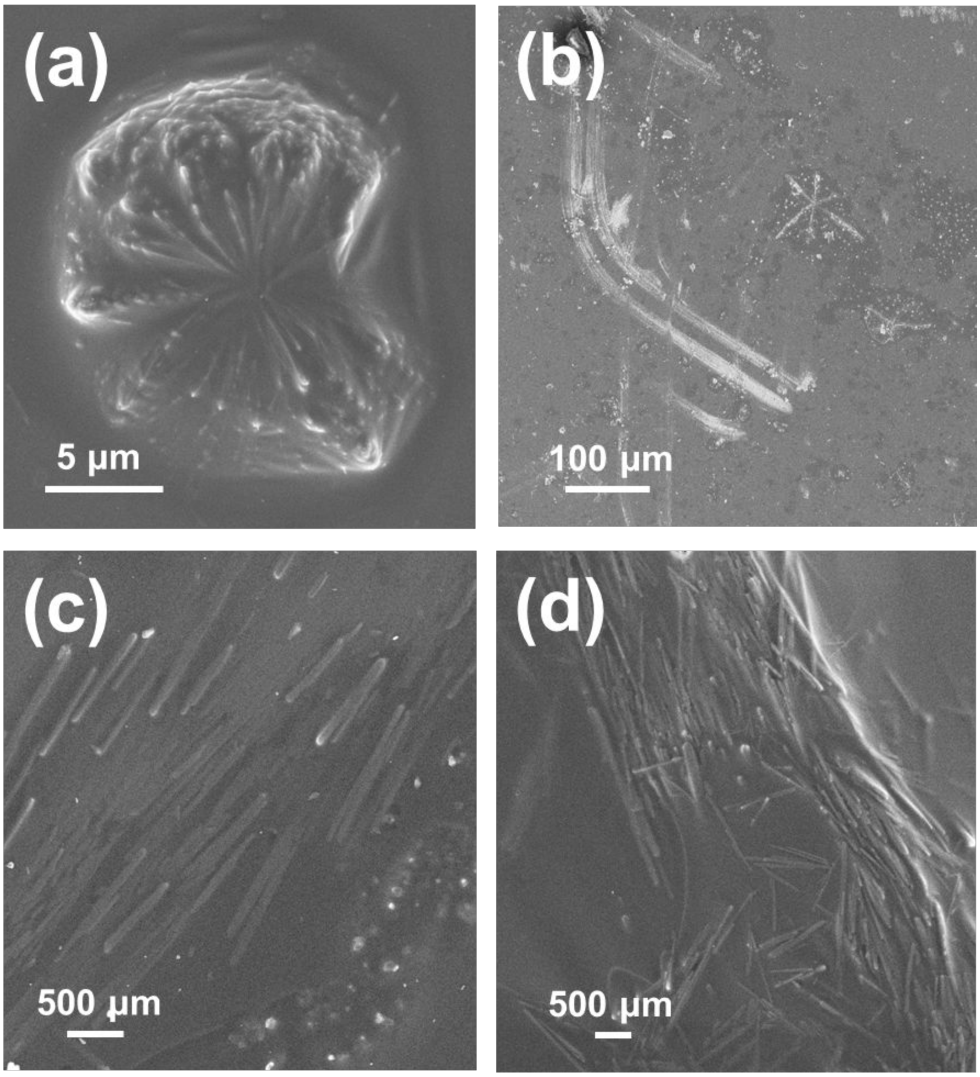

Figure S2. SEM micrographs of poorly defined microstructures formed from CREKA solutions at 4 ºC using the following conditions: (a) 0.01, (b) 0.1 and (c) 1 mg/mL peptide concentration at pH 4, and (d) 0.1 mg/mL peptide concentration at pH 7.

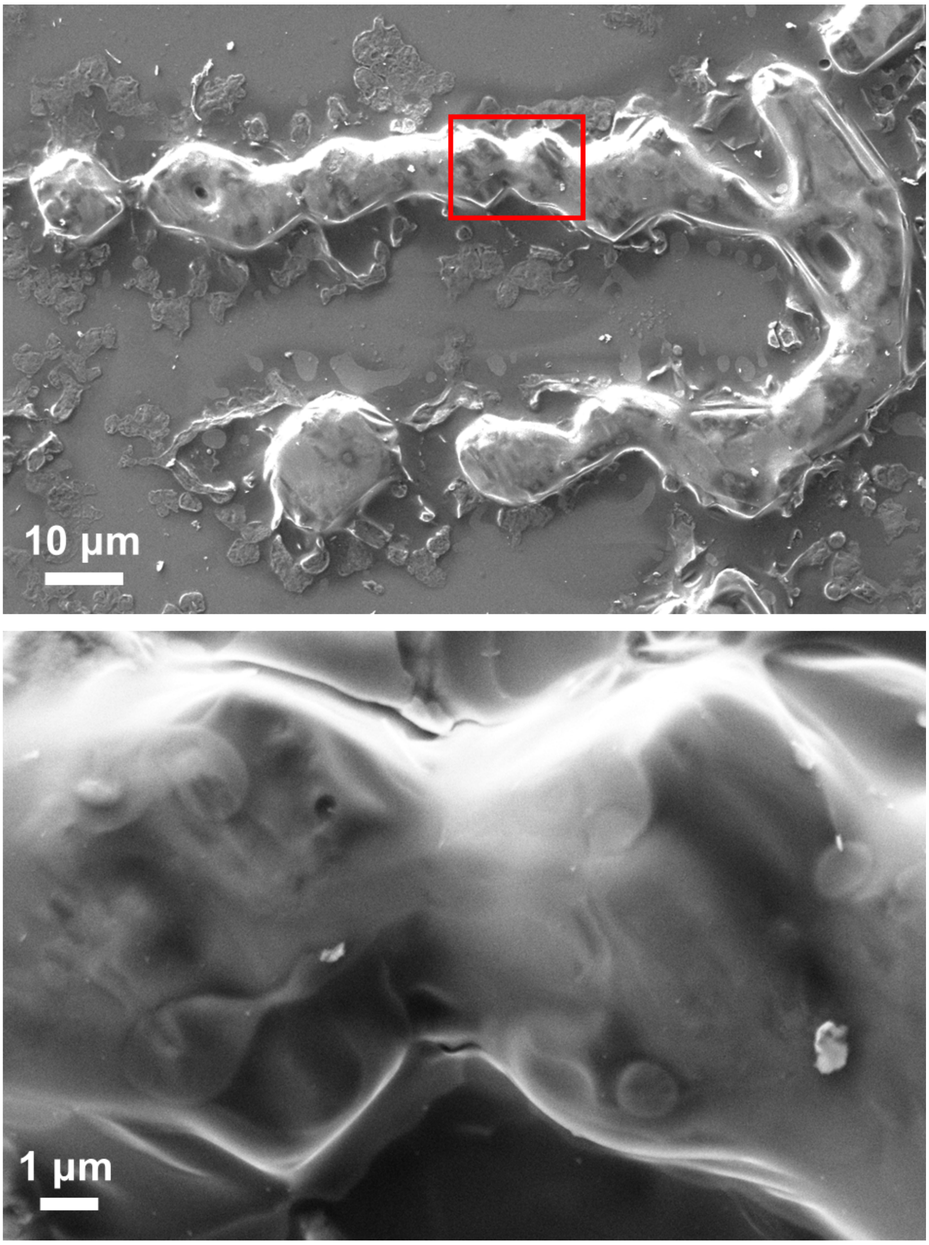

Figure S3. SEM micrographs of structures derived from 0.1 mg/mL CRE^{N}KA solutions at pH 7 and 4 ºC. Micrographs, which were recorded when the solvent had not been completely evaporated, show the contact and merging of preformed particles.

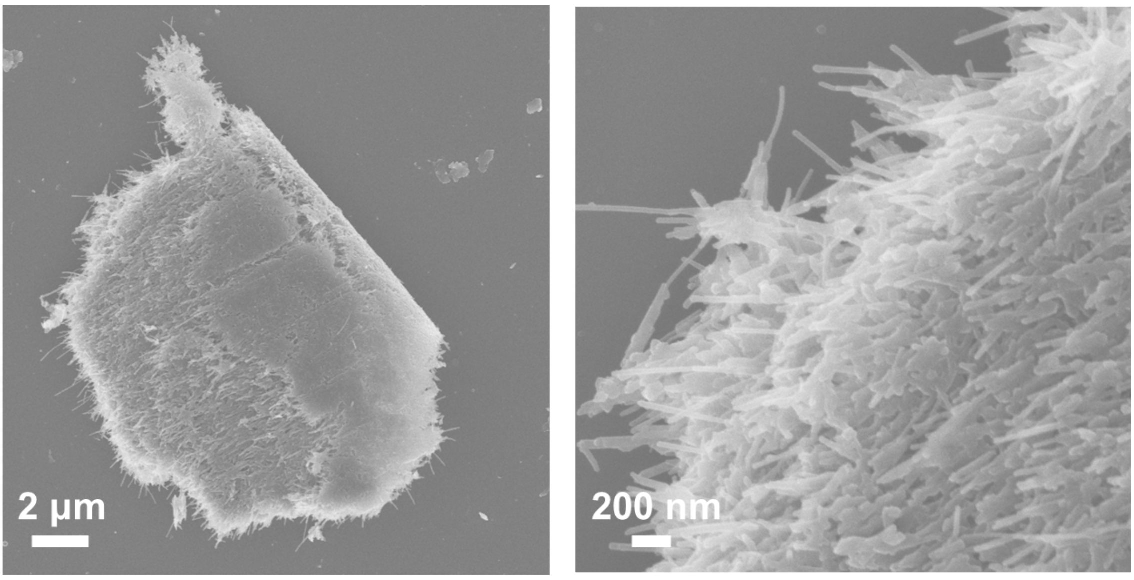

Figure S4. SEM micrographs of irregular particles made of nanofibers obtained for 2.0 mg/mL CRE^{N}KA solutions at pH 7.
